# Microenvironmental control of stem cell fate in intestinal homeostasis and disease

**DOI:** 10.1002/path.4563

**Published:** 2015-06-15

**Authors:** Sujata Biswas, Hayley Davis, Shazia Irshad, Tessa Sandberg, Daniel Worthley, Simon Leedham

**Affiliations:** ^1^ Gastrointestinal Stem Cell Biology Laboratory, Wellcome Trust Centre for Human GeneticsUniversity of OxfordRoosevelt DriveOxfordUK; ^2^ Translational Gastroenterology Unit, Experimental Medicine Division, Nuffield Department of Clinical MedicineJohn Radcliffe HospitalHeadingtonOxfordUK; ^3^ Molecular and Population Genetics Laboratory, Wellcome Trust Centre for Human GeneticsUniversity of OxfordRoosevelt DriveOxfordUK; ^4^ South Australian Health and Medical Research InstituteUniversity of AdelaideAdelaideSouth AustraliaAustralia

**Keywords:** colon, neoplasia, chronic inflammation

## Abstract

The conventional model of intestinal epithelial architecture describes a unidirectional tissue organizational hierarchy with stem cells situated at the crypt base and daughter cells proliferating and terminally differentiating as they progress along the vertical (crypt–luminal) axis. In this model, the fate of a cell that has left the niche is determined and its lifespan limited. Evidence is accumulating to suggest that stem cell control and daughter cell fate determination is not solely an intrinsic, cell autonomous property but is heavily influenced by the microenvironment including paracrine, mesenchymal, and endogenous epithelial morphogen gradients. Recent research suggests that in intestinal homeostasis, stem cells transit reversibly between states of variable competence in the niche. Furthermore, selective pressures that disrupt the homeostatic balance, such as intestinal inflammation or morphogen dysregulation, can cause committed progenitor cells and even some differentiated cells to regain stem cell properties. Importantly, it has been recently shown that this disruption of cell fate determination can lead to somatic mutation and neoplastic transformation of cells situated outside the crypt base stem cell niche. This paper reviews the exciting developments in the study of stem cell dynamics in homeostasis, intestinal regeneration, and carcinogenesis, and explores the implications for human disease and cancer therapies. © 2015 Authors. The Journal of Pathology published by John Wiley & Sons Ltd on behalf of Pathological Society of Great Britain and Ireland.

## Introduction

The intestinal epithelium is a paradigm for the systems governing cellular renewal in the epithelial tissues that line our body. The gut mucosa has evolved to selectively absorb nutrients and water whilst protecting the body from the toxic contents of the gut lumen. This protection is afforded by the continuous renewal of the epithelium along the vertical (crypt‐to‐luminal surface) axis of the intestine, allowing migrating, differentiated epithelial cells that have been exposed to genotoxic luminal contents to apoptose and be shed into the gut lumen. Intestinal epithelial turnover depends on a self‐renewing stem cell population with rapid expansion of stem cell progeny followed by differentiation of the daughter cell population, all within the 5–7 days that it takes for a cell to migrate from the crypt base to the luminal surface. The hierarchical and stereotypical architecture of the intestinal epithelium makes it an attractive model for the study of tissue‐specific stem cells, alongside the mechanisms that control cell fate determination – processes that are fundamental for homeostasis and frequently deranged in carcinogenesis.

Experimental approaches to study these mechanisms are reliant on the ability to accurately identify stem cells and then trace the fate of their progeny. Meticulous, pioneering work in the 1970s used radiation sensitivity and surrogate markers to identify two distinct populations of cells with stem‐like properties – crypt base columnar (CBC) cells 1 and label‐retaining cells at position +4 relative to the base of the intestinal crypt 2. It was a further three decades before transgenic fluorescent labelling of molecules selectively expressed by these cell populations permitted the accurate identification and tracing of murine stem cells and their progeny. Work in the field continues to evolve. Continuous intravital imaging has allowed the temporal observation of stem cell dynamics, whilst the manipulation of the intestinal microenvironment indicates that stem cell function and daughter cell fate determination is not exclusively an intrinsic, cell autonomous property.

This review will explore some of the recent advances in our understanding of the control of the intestinal stem cell and daughter cell fate determination in intestinal homeostasis and assess the implications for colorectal carcinogenesis.

## Intestinal architecture, stem cells, and morphogenic control

**Figure 1:**
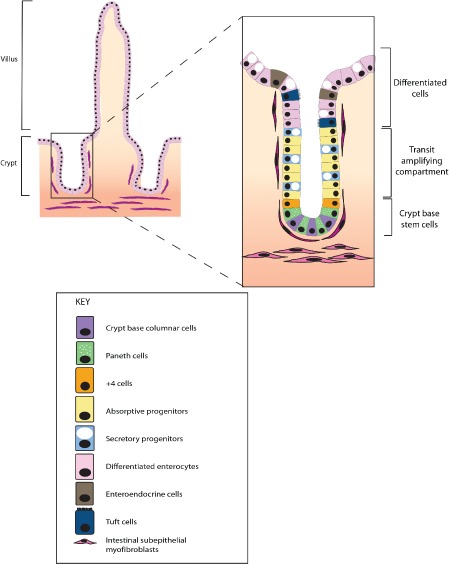
Intestinal crypt architecture and cell types. The intestinal crypt is the basic functional unit of the gut. In the small intestine, several crypts contribute to finger‐like projections called villi. In homeostasis, the stem cells (crypt base columnar and +4 cells) are restricted to the crypt base stem cell niche. Immediate stem cell progeny divide rapidly in the bottom half of the crypt, called the transit amplifying zone. Terminal differentiation occurs in the upper part of the crypt, with fully differentiated cells eventually being shed into the intestinal lumen. Under homeostatic conditions in the mammalian gut, transit along the crypt–luminal axis takes 5–7 days.

The luminal surface of the intestine is composed of columnar epithelial mucosa with glandular invaginations called crypts, the basic functional unit of the gut. In the small intestine, several of these crypts contribute epithelium to an individual villus. The intestinal epithelium is continually shed and replaced, and this capacity for self‐renewal is supported by intestinal stem cells at the crypt base. Immediate stem cell progeny undergo rapid proliferation in the bottom third of the intestinal crypt to expand the population of progenitor cells required for epithelial turnover. This proliferative region is called the transit amplifying cell compartment. Following cell proliferation, daughter cells differentiate as they pass along the crypt–villus axis, with five main specialized epithelial lineages arising from post‐mitotic, terminal differentiation. Enterocytes absorb nutrients and make up the majority of the epithelial cells. Goblet cells produce mucus to provide a protective barrier. Enteroendocrine cells secrete gastrointestinal hormones. Despite being the largest endocrine system in the body, these cells make up less than 1% of the epithelium. Paneth cells, although differentiated, reside at the crypt base and secrete antimicrobial peptides as well as growth factors to maintain the stem cell niche. Tuft or brush cells are a secretory cell lineage, so‐named by the brush of microvilli on their apical surface. They secrete prostaglandin precursors, are thought to mediate the interaction with the enteric nervous system, and are identified by expression of *DCLK1 (doublecortin‐like kinase 1)* (Figure 1).

## Stem cell identification

Stem cells are defined functionally by their potential for self‐renewal and multipotency – in the gut, this effectively means the capacity to differentiate into all of the intestinal epithelial cell types listed above 3. Recent advances in biotechnology have led to a surge of studies that have characterized the properties and interactions of putative intestinal stem cell populations in vivo 4, 5. Transgenic activation of heritable fluorescent labels in cells expressing candidate stem cell markers allows accurate cell fate mapping and progeny lineage tracing over time, to satisfy the defining stem cell characteristics of self‐renewal and multipotency. However, this technique requires prior knowledge of the marker to be tested and cannot assess the stem cell capacity of cells not expressing the candidate gene. Stem cell markers generally identify a population of cells enriched for stem cell properties, and marker expression alone does not substitute for functional stem cell definition. Lineage tracing remains the gold standard for assessing stem cell function and has resulted in significant advances in the characterization of the CBC cells 1 and the label‐retaining cells in the +4 crypt position 2.

By focusing on Wnt signalling as the main pathway controlling stem cell function, the Clevers group established a panel of Wnt target genes and used in situ hybridization to identify those genes with restricted crypt base expression 6, 7. Transgenic mice were generated to lineage trace cells expressing the Wnt target gene Lgr5 (leucine‐rich‐repeat‐containing G‐protein‐coupled receptor 5), which is restricted to the crypt base columnar cells 8. Over a 60‐day period, all epithelial cell types were produced from the Lgr5‐positive transgenically labelled crypt base columnar cells. Lgr5 has subsequently been validated as a bona fide stem cell marker in the small intestine, colon 9, stomach pylorus 10, and other organs such as the hair follicle 11.

In contrast to the Lgr5 cells which divide about once a day, the cells in the +4 position of the crypt, also defined by the property of label retention, are quiescent and slowly cycling. Several cell markers with expression patterns that overlie this position have been described 12, 13, 14. Using a tamoxifen‐inducible Bmi1 Cre recombinase mouse, Sangiorgi et al demonstrated lineage tracing from cells at the +4 position 12. Further work showed that Bmi1 is in fact expressed broadly throughout the crypt, diminishing this as a specific stem cell marker 15, 16, 17. Rather than relying on putative marker expression, Buczacki et al focused on cells with functional label retention properties and in a series of elegant experiments, demonstrated that these cells are committed secretory cell precursors that retain the capability of returning to stem cell function in the event of intestinal damage and regeneration 18. Numerous other markers have been used to identify populations that are enriched for cells capable of lineage tracing in the intestine including Tert, Hopx, Lrig1, Sox9, Ascl2, Olfm4, Prom1, and Rnf43 15, 19, 20, 21, 22.

## The stem cell niche and morphogenic control of cell fate determination

Regulation of normal intestinal stem cells occurs within a discrete microenvironment confined to the crypt base, known as the stem cell niche. This consists of epithelial and mesenchymal cells and extracellular substrates which favour the existence of a stem cell in its undifferentiated state. It provides an optimal microenvironment for the production of differentiated progeny by the paracrine secretion of growth factors, cytokines, and morphogens (soluble molecules produced by a restricted region of a tissue that form an activity gradient away from its source). The phenotypic response of a cell is determined by its position within these concentration gradients. Key constituents of the niche include Paneth cells and pericryptal fibroblasts, which provide critical factors for the survival of crypt base stem cells such as Wnt3a, epidermal growth factor (EGF), and bone morphogenetic protein (BMP) antagonists 23.

**Figure 2:**
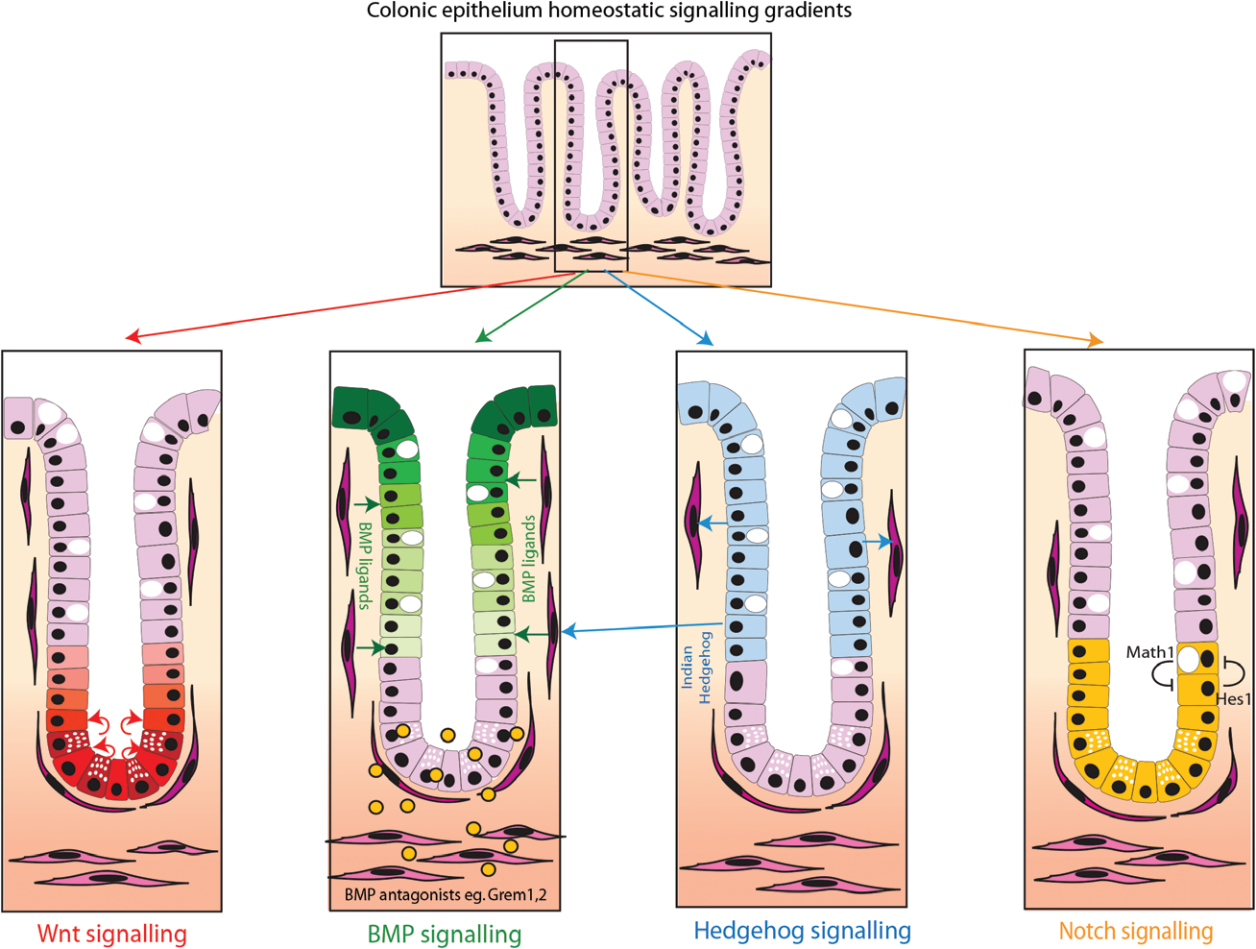
Key morphogen signalling pathway gradients in intestinal homeostasis. Intestinal homeostasis and cell fate determination are maintained by a complex interaction of polarized epithelial and mesenchymal morphogen signalling pathways. Major pathways include (A) Wnt signalling. Wnt ligands, secreted from the intestinal subepithelial myofibrobasts and the Paneth cells, act predominantly at the base of the crypt (red cells) to maintain stem cell function and transit amplifying cell proliferation; (B) Bone morphogenetic protein signalling. BMP ligands are produced predominantly by the mesenchymal cells, although BMP2 is also expressed by epithelial cells. BMP represses Wnt signalling 26 and promotes cell differentiation and apoptosis, acting predominantly in the upper part of the crypt and villus (green cells). There is reduced BMP activity at the base of the crypt, partly through diffusion gradients of the ligands but also secondary to the restricted expression of ligand‐sequestering BMP antagonists from the sub‐crypt myofibroblasts; (C) Hedgehog signalling. Indian Hedgehog (IHH) is the main ligand and is expressed by epithelial cells in the upper part of the crypt (blue cells). IHH acts upon and maintains the myofibroblasts. This has a secondary effect on the epithelium through promotion of BMP ligand expression; (D) Notch signalling. Notch regulates cell fate through cell‐to‐cell contact in the stem cell and transit amplifying zone at the base of the crypt (yellow cells). Notch activation via Hes1 transcription factor and lateral inhibition via Math1 transcription factor regulate enterocyte or secretory cell fate, respectively.

Adult stem cell and daughter cell fate determination is controlled by the same signalling pathways that regulate embryonic stem cell function during development. In the adult, these pathways are stringently controlled with complex interactions used to restrict pathway activity and response to the appropriate cell compartment. Mesenchymal and epithelial‐derived pathways result in polarized gradients that regulate stemness, cell proliferation, differentiation, and apoptosis as cells progress along the intestinal vertical axis. Important pathways include Wnt, BMP, Hedgehog, and Notch signalling (Figure 2).

### Wnt signalling

This highly conserved signalling pathway involved in cell fate specification is expressed in species ranging from *Drosophila* to man. Beta‐catenin is the key link in the pathway. Usually anchored to the basement membrane, intracellular β‐catenin is minimized by phosphorylation and ubiquitin‐proteasome degradation of its complex with adenomatous polyposis coli (APC). Binding of Wnt to Frizzled receptors inhibits degradation of β‐catenin, so increasing its cytosolic level. This results in transcription of target genes driving proliferation. The non‐canonical Wnt pathway, which does not involve β‐catenin, is also essential for epithelial proliferation. In the intestine, Wnt signalling is mainly restricted to the stem and proliferative zones in the bottom third of the crypt, where it is involved in maintenance of stem cell function and driving transit amplifying cell proliferation.

### Bone morphogenetic protein (BMP)

BMP signalling is part of the TGFβ (transforming growth factor β) superfamily and phosphorylates Smad proteins for signal transduction. BMP signalling has a pivotal role in intestinal development and is required for the control of intestinal stem cell replication. It is also needed for terminal differentiation of mature intestinal cells 24. The BMP ligands are secreted from both epithelial and mesenchymal cells but act mainly on the epithelial compartment through epithelial cell expression of BMP receptors 25. BMP represses Wnt signalling, so opposing gradients exist, with BMP signalling highest in the cells at the luminal surface 26. BMP signalling at the crypt base stem cell niche is carefully regulated by the expression of restricted gradients of exclusively mesenchymally expressed ligand‐sequestering BMP antagonists such as *Gremlin1*, *2*, and, to a lesser extent, *Noggin*.

### Hedgehog signalling

Hedgehog controls tissue polarity. Indian hedgehog is the main Hedgehog protein expressed in the intestine and is secreted in a paracrine manner by differentiated epithelial cells to act on mesenchymal cells. It maintains homeostasis of mesenchymal cells and regulates epithelial cell proliferation through negative feedback to proliferating crypt base columnar cells by increasing BMP signalling 27.

### Notch signalling

Notch interacts with Delta and Jagged ligands and signals through cell–cell contact to regulate post‐mitotic differentiation into the different cell lineages. It thus regulates cell fate decision between absorptive and secretory cell types. Notch signalling is also involved in the control of stem cells and transit amplifying cell division 28, 29. High levels of Notch inhibit transcription of the *Math1* gene via the transcriptional repressor *Hes1*. Lateral inhibition in this cell‐to‐cell signalling pathway means that adjacent cells are driven towards different fates, resulting in a ‘salt and pepper’ distribution of progenitor cells committed to enterocyte and secretory lineages 30.

## Stem cell dynamics in homeostasis

It is increasingly recognized that stem cell function is not an intrinsic, cell autonomous property and that stem cell fate is not fixed, even within the confines of the stem cell niche. Elegant work by the Winton and Clevers groups has shown that murine intestinal stem cells form an equipotent population that undergoes stochastic clone extinction, compensated by expansion and replacement by a neighbouring cell. This random enlargement and contraction of clones, known as neutral drift dynamics, can lead to stochastic clonal extinction or niche succession, where one clone expands to fill the entire niche 31, 32. Similar neutral drift dynamics are seen in human colon using mtDNA mutations as clonal markers 33. Recent ground‐breaking work by Ritsma *et al* 5 used surgical implantation of abdominal imaging windows and continuous intravital imaging to temporally observe fluorescently labelled stem cell dynamics *in vivo*. Quantitative analysis of individual clonal lineages showed that ‘central cells’ at the crypt base, optimally located near niche‐derived signals, had a survival advantage over ‘border cells’ in the upper part of the niche. Compared with their centrally located counterparts, border cells were more likely to be displaced into the transit‐amplifying compartment, lose stem cell properties, and progress along the crypt–villus escalator. This indicates that there is a spectrum of stem cell function even within cells expressing the *Lgr5* marker and that these cells transit reversibly between states of variable competence influenced by proximity to niche‐derived signals. Somatic events such as *Apc* and *Kras* mutations that bestow a selective advantage can bias a stem cell towards clonal expansion within the niche, although Vermeulen *et al* 34 showed that this is not necessarily inevitable. Interestingly, other mutations such as *Tp53* inactivation only resulted in a selective advantage and resultant niche succession in an inflammatory microenvironment, which highlights the critical interplay between genotype and environmental context in disrupted homeostasis and colorectal carcinogenesis.

## Disrupted homeostasis and intestinal regeneration

The intestinal epithelium is the first line of defence in a hostile environment and is subject to frequent chemical, microbiological, and immunological mediated mucosal damage. Many of the evolutionarily conserved pathways that control cell fate determination in the intestine are also involved in the physiological response to damage and intestinal regeneration. Regulated disruption of these epithelial, stromal, and immunological morphogen gradients with resultant change in epithelial stem cell function is a key feature of the response to damage and underpins the regenerative and reparative capacity of the intestinal epithelium. Lineage tracing studies using different stem cell markers have defined a number of cell populations enriched for stem cell potential/activity within the base of the crypt and dynamic studies using the most established markers have shown considerable variability in stem cell competence even during intestinal homeostasis. Recent work using specific killing of a single marker‐defined cell population or more widespread damage has helped to assess stem and daughter cell dynamic changes when homeostasis is disrupted.

Within the stem cell niche, genetic ablation of a specific stem cell causes a reserve pool to step up to prevent an overall phenotypic change 35. Thus, diphtheria toxin receptor (DTR)‐mediated ablation of *Lgr5+* cells in mice caused no phenotypic change because of the compensatory effects of an expansion in the *Bmi1+* cell population 35. This indicates the bidirectional interconversion between +4 cells and the crypt base columnar cells. Induction of chemical colitis and radiation damage provokes a more indiscriminate intestinal injury, associated with a mucosal inflammatory response. Recent work has shown that this disruption of the crypt microenvironment can provoke a return to stem cell function from cells that have ostensibly exited the stem cell niche. Lineage tracing from *Lgr5*‐negative, notch ligand *Delta like 1*‐expressing secretory progenitors resulted in full crypt–villus lineage tracing following intestinal radiation 36. Metcalfe *et al* showed that intestinal architecture can regenerate if either the stem cell or the progenitor zone remains intact following injury; however, if both cell populations are damaged/ablated simultaneously, the intestinal architecture is irreversibly perturbed 37.

These findings oppose our dogmatic understanding of a strict unidirectional tissue organizational hierarchy in the intestine and raise questions over the limits of intestinal cell plasticity. If progenitor cells are capable of dedifferentiation and return to stem cell function following injury, is the fate of fully differentiated cells fixed? Westphalen *et al* used a *doublecortin‐like kinase1* transgenic mouse to label and trace the fate of mature tuft cells in the murine colon. Surprisingly, they showed that these quiescent post‐mitotic, differentiated cells survived outside of the crypt base for many months, resisting the stream of enterocytes and goblet cells passing along the crypt–villus escalator. Rare lineage tracing events in the stomach and intestine were seen from transgenically labelled tuft cells and genetic ablation of these cells impaired the ability of the intestine to respond to radiation‐ or colitis‐induced damage 38.

Thus, it appears that regulated, temporary disruption of morphogen gradients in a damaged mucosa is essential to alter stem cell dynamics, dedifferentiate progenitor and even some differentiated cells, and promote epithelial cell migration and proliferation to effect epithelial restitution 39. However, the same characteristics are key attributes of malignant cells; thus, failure of restoration of tight homeostatic control in intestinal inflammation or pathological disruption of morphogen gradients can result in the initiation of neoplasia.

## Microenvironmental disruption and the initiation of neoplasia

The cellular origin of colorectal neoplasia has been controversial, centred on the mechanism of clonal expansion in human colorectal precursor lesions. The ‘top down’ model was based on the observation that some early adenomatous lesions develop on the luminal surface without any obvious contact to the crypt base and proposed an intercrypt location for the stem cell niche. The ‘bottom up’ model was founded on the more conventional crypt base stem cell compartment and an assumed unidirectional tissue organizational hierarchy. This model proposed that dysplasia arose in the stem cells at the crypt base, with subsequent spread of dysplastic progeny along the length of the crypt 40. This model was strongly supported by the induction of progressive intestinal tumourigenesis as a result of the selective introduction of Wnt‐activating mutations into *Lgr5+* crypt base columnar cells, which was a stark contrast to the small non‐progressive phenotype provoked by introducing the same mutations into non‐stem cells 41. Although the bottom up model fits for colorectal carcinogenesis initiated by a Wnt‐disrupting genetic mutation in a crypt base stem cell, several recent publications have shown that a top down model of tumour histogenesis may fit other subtypes of cancer where microenvironmental disruption, alongside epithelial genetic change, plays a key role in lesion initiation.

**Figure 3:**
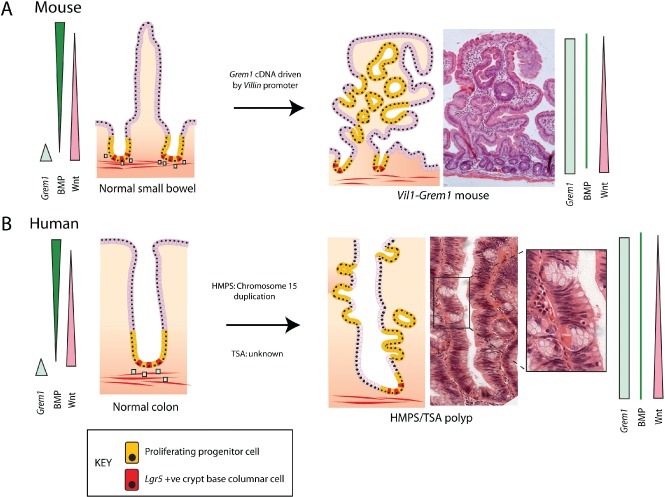
Compartmental expression switch of GREM1 expression initiates neoplasia from an expanded progenitor cell population. Aberrant epithelial expression of the BMP antagonist GREM1 disrupts the polarized BMP gradient and dysregulates cell fate determination along the crypt–villus axis of the intestine. This results in the expansion of a proliferating progenitor population which forms ectopic crypt foci. (A) Mouse. In the mouse, ectopic crypts are seen on the villi of the small intestine and dysplasia arises from within these intravillus lesions. (B) Human. In human hereditary mixed polyposis syndrome (HMPS) and sporadic traditional serrated adenoma (TSA) lesions, ectopic crypts can be seen developing orthogonally to the axis of the crypt. Coloured bars represent morphogen gradients in the normal and pathological states. Blue squares represent physiological Grem1 expression from pericryptal myofibroblasts. CBC stem cells are red and progenitor cells are yellow.

Schwitalla *et al* used mouse models to demonstrate that activated nuclear factor‐kappa B‐induced mucosal inflammation, in combination with constitutive epithelial Wnt signalling transgenically targeted to the non‐stem cell population, could induce dedifferentiation of *Lgr5*‐negative cells situated outside of the crypt base stem cell niche. Critically, they also showed that it was these dedifferentiated villus cells that gave rise to the tumours that rapidly arose in these animals 42. Transgenic targeting of *Apc* mutations to long‐lived, quiescent tuft cells had no phenotypic effect in a normal environmental context, but gave rise to aggressive, poorly differentiated carcinoma when intestinal inflammation was induced with dextran sodium sulphate, highlighting the dependence of both cell‐intrinsic (genetic) and cell‐extrinsic (microenvironmental) factors in promoting carcinogenic initiation in this situation 38. We have recently shown that this environmental change is not restricted to induction of mucosal inflammation. Pathological disruption of the homeostatic morphogen balance is also capable of disrupting cell fate determination, resulting in both murine and human intestinal tumourigenesis. Human hereditary mixed polyposis syndrome (HMPS) is an autosomal dominant condition named for the distinctive morphology of the polyps, with individual lesions exhibiting mixed adenomatous crypts, dilated cysts, and epithelial serration. Characteristically, HMPS polyps develop small ectopic crypts that grow orthogonally to the crypt–villus axis (ectopic crypt foci, ECF). HMPS is caused by a 40 kb duplication upstream of the BMP antagonist *GREM1*, which results in a compartmental expression switch – from a restricted mesenchymal gradient to ectopic gene expression throughout the epithelium 43. A transgenic mouse modelling this compartmental change in *Grem1* expression developed an HMPS phenotype with mixed morphology polyps arising from ectopic crypts. We demonstrated that this disruption of polarized homeostatic BMP signalling gradients led to the expansion of an *Lgr5*‐negative progenitor cell population into ectopic crypts. These ECF progenitor cells actively proliferate and accumulate somatic mutations, eventually resulting in intestinal neoplasia arising outside the crypt base stem cell niche (Figure 3). Interestingly, the ectopic crypt phenotype is also shared by human sporadic traditional serrated adenomas, lesions with a hitherto unknown pathogenesis. We have shown that these lesions also initiate dysplasia from an expanded population of progenitor cells within ectopic crypt foci and that they are similarly caused by aberrant epithelial expression of *GREM1*. Traditional serrated adenomas can thus be considered the sporadic counterpart of HMPS polyps 44.

## Genetic and environmental interaction and CRC heterogeneity

**Figure 4:**
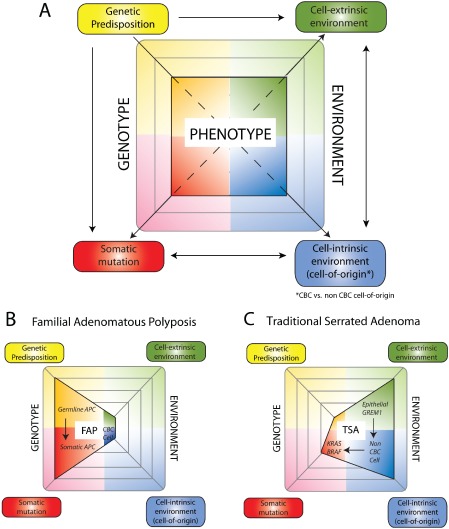
Genetic and environmental influences variably contribute to cancer phenotype in different colorectal cancer subtypes. A key feature of Darwinian evolutionary theory is that cancer phenotype is determined by a combination of genetic and environmental influences. (A) We propose an update of this important maxim by dividing genotype into genetic predisposition and somatic mutation, and environment into cell‐intrinsic (cell‐of‐origin) and cell‐extrinsic environment. There is considerable interaction between these different influences, eg the cell of origin selects for an optimal somatic mutation (arrows). The variety of cancer phenotypes can be related to the variable importance of these four different influences in tumour development. (B) Familial adenomatous polyposis (FAP). In FAP, germline APC mutation in epithelial stem cells predominantly drives tumourigenesis without the requirement for an altered cell‐intrinsic or cell‐extrinsic microenvironment. (C) Traditional serrated adenoma (TSA). In contrast, in sporadic TSAs, the aberrant morphogen environment provoked by epithelial GREM1 expression leads to cancer initiation from cells outside of the crypt base. This selects for optimal somatic mutations that differ from conventional Wnt‐driven carcinogenic pathways. CBC cell = crypt base columnar cell.

The work described in this review highlights the impact of the microenvironmental context on intestinal epithelial stem cell function both in health and in disease. With respect to intestinal tumourigenesis, predominantly thought to be driven by genetic mutation in epithelial stem cells, this provides a timely reminder of the evolutionary maxim that phenotype is a consequence of the interaction of both genotype (which can be subdivided into genetic predisposition and somatic mutation) and environment (subdivided into cell‐intrinsic and cell‐extrinsic environment) (Figure 4). The relative importance of these factors may vary within the increasing number of different tumour subtypes, but the cancer phenotype is determined by a variable and interacting contribution from all of these influences, not solely from acquired genetic mutation.

The implications of this conceptual shift can be interpreted in light of the recent evidence demonstrating the molecular heterogeneity of colorectal cancer. Several recent publications have sought to classify and subtype colorectal cancer based on (epi)genetic mutation and gene expression pattern 45, 46, 47. These molecular profiles correlate with variable prognosis and response to therapy and appear to reflect considerable differences in tumour subtype biology that cannot be predicted from their acquired epithelial genetic mutation burden alone. Two very recent publications demonstrate the important contribution of the cancer stroma to the variable molecular signature seen in different CRC subtypes. Both studies independently show that each of the stromal tissue elements (cancer‐associated fibroblasts, infiltrating immunocytes, and endothelial cells) contributes more towards poor prognosis molecular signatures than the cancer epithelium itself 48, 49. In these poor prognosis tumours, stromal molecular signatures were also associated with a poor response to radiotherapy in rectal cancer, indicating the important influence of the tumour microenvironment on cancer epithelial cell behaviour and response to treatment.

## Implications

The notion of a unidirectional tissue organizational hierarchy in the intestine has provided a useful conceptual framework to support our understanding of the location and function of intestinal stem cells, the determination of daughter cell fate, and the cell of origin of colorectal cancer. Recently, the ability to selectively manipulate the intestinal microenvironment and the increasing sophistication of the transgenic animal tools used to assess stem cell dynamics have shown that this model requires re‐evaluation. Stem cells are defined functionally by the characteristics of self‐renewal and multi‐lineage potential, not as individual cells determined by the expression of one or more markers. The work in this review highlights that the stem cell pool should be thought of as a functional collective, comprising a dynamic and heterogeneous population of cells with variable stem cell competence imposed by position within the niche and microenvironmental morphogen context.

Colorectal cancer remains a paradigm for the cancer stem cell model, which proposes that tumours are maintained by a small population of tumourigenic, self‐renewing cancer stem cells that are at the apex of a cellular hierarchy, not dissimilar to that seen in normal tissue. The bulk of the tumour is thus made up of non‐clonogenic daughter cells that have initiated organ‐specific differentiation programmes and have limited tumourigenic and metastatic potential. There is evidence to support this assertion: single colorectal cancer cells are capable of multi‐lineage differentiation 50, 51 and colorectal cancers are histopathologically diverse, with different regions exhibiting varying degrees of differentiation 52. There is, however, compelling evidence and increasing acceptance in the cancer stem cell field that stemness is a dynamic state with cancer stem cell plasticity multifactorially driven by cell‐intrinsic factors, microenvironmental signals, and evolutionary pressures. Cancer stem cell plasticity bestows a considerable evolutionary advantage on a tumour by allowing it to adapt to a changing environment and selective pressures imposed by tumour context, immune activation, and chemotherapy. Furthermore, stem cell expression signatures in tumours correlate with poor prognosis and metastasis 53, 54. The genetic, epigenetic, and population level factors affecting cancer stem cell plasticity are outside the context of this article but are comprehensively covered in other reviews 55, 56.

In contrast to the exquisitely controlled and balanced morphogen signalling seen in intestinal homeostasis, the cancer stem cell microenvironment is a disorganized and chaotic signalling mix, originating from cancer‐associated fibroblasts, infiltrating immunocytes, and endothelial cells. Working with primary CRC cultures, Vermeulen *et al* identified myofibroblast‐derived hepatocyte growth factor (HGF) as an activator of β‐catenin‐dependent transcription and resultant epithelial cancer stem cell clonogenicity 57 and this acts alongside *osteopontin* and *SDF1* to impose a cancer stem cell phenotype on CRC progenitor cells 58. Infiltrating CD4+ T cells secrete *IL‐22*, which acts through activation of *STAT3* signalling and *DOT1L* methyltransferase to influence cancer stem cell stemness. Neutralization of *IL‐22* signalling can attenuate and even reverse an inflammation‐driven mouse model of colorectal cancer 59. Treatment of tumours also has an effect on the microenvironment. Chemotherapy can remodel the tumour microenvironment by increasing the number of cancer‐associated fibroblasts that secrete cytokines such as IL‐17A to promote tumourigenesis and resistance to therapy 60.

**Figure 5:**
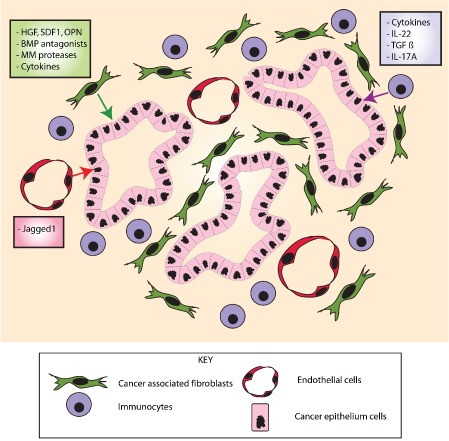
Stromal signalling pathways affecting epithelial cell stem cell function in the cancer microenvironment. Different cancer stromal elements express numerous factors that act upon the epithelium to modulate epithelial cancer stem cell function. The cancer‐associated fibroblasts express BMP antagonists and factors such as hepatocyte growth factor (HGF) and osteopontin (OPN). Tumour‐infiltrating immune cells secrete cytokines such as IL‐22 and IL‐17, and endothelial cells interact with the epithelium via expression of Jagged1, regulating the Notch pathway.

Even tumour‐associated endothelial cells have recently been shown to have an effect on the cancer stem cell phenotype by paracrine secretion of the Notch ligand *Jagged1* 61 (Figure 5). Our work in human tumours shows that aberrant epithelial and/or mesenchymally derived *GREM1* expression is associated with colorectal cancer subtypes that respond poorly to current chemotherapeutic regimes and have a dismal outcome 45. Given the demonstrated role of *GREM1* in imposing a stem cell phenotype on cells outside the crypt base, it seems plausible that the poor treatment response may be secondary to an effect of *GREM1* on cancer stem cell plasticity. The identification of disrupted signalling pathways that influence cancer stem cell plasticity could open up therapeutic targets that aim to redress the signalling balance in tumours. Resetting the morphogen balance in tumours could reduce the speed or effectiveness of CSC dynamic change in response to chemotherapeutic selective pressures, allowing current drugs to kill cancer stem cells more effectively.

## Conclusion

As experimental techniques improve, our understanding of the position, fate, and role of intestinal stem cells in homeostasis and carcinogenesis is evolving. The intestinal stem cell pool is a dynamic population of cells whose fate is heavily influenced by the microenvironmental signals received from surrounding supporting tissue. The epithelial response to disruption of these signals is a critical feature of the intestine's ability to respond to injury, yet signalling pathway dysregulation is also a key feature in the initiation of cancer. Teasing apart how to manipulate disrupted signalling in tumours without affecting the same pathway's role in tissue homeostasis is the critical challenge for future drug design. To understand and then target the factors that discriminate the neoplastic from the normal stem cell niche will be the next step.

### Author contribution statement

SB and SL wrote the article. HD, SI, TS, and DW reviewed and edited the manuscript.
